# Familial patterning and prevalence of male androphilia among Istmo Zapotec men and *muxes*

**DOI:** 10.1371/journal.pone.0192683

**Published:** 2018-02-21

**Authors:** Francisco R. Gómez, Scott W. Semenyna, Lucas Court, Paul L. Vasey

**Affiliations:** Department of Psychology, University of Lethbridge, Lethbridge, Alberta, Canada; Universita degli Studi di Pisa, ITALY

## Abstract

Male androphilia (i.e., male sexual attraction to other adult males) is known to cluster within families. Some studies demonstrate that male androphilia clusters in both the paternal and maternal familial lines, whereas other studies demonstrated that it clusters only in the latter. Most of these studies were conducted in Euro-American populations where fertility is low and the sexual orientation of male relatives can sometimes be difficult to ascertain. These two factors can potentially confound the results of such studies. To address these limitations, we examined the familial patterning of male androphilia among the Istmo Zapotec of Oaxaca, Mexico––a high fertility, non-Euro-American population where androphilic males are known locally as *muxes*, a third gender category. The Istmo Zapotec recognize two types of *muxes*––*muxe gunaa* and *muxe nguiiu––*who typify the transgender and cisgender forms of male androphilia, respectively. We compared the familial patterning of male androphilia between *muxe gunaa* and *muxe nguiiu*, as well as between gynephilic men and *muxes* (both cisgender and transgender forms combined). Istmo Zapotec *muxe gunaa* and *muxe nguiiu* exhibit similar familial patterning of male androphilia. Overall, *muxes* were characterized by significantly more *muxe* relatives than gynephilic men. This familial patterning was equivalent in both the paternal and maternal lines of *muxes*. The population prevalence rate of male androphilia was estimated to fall between 3.37–6.02% in the Istmo Zapotec. This is the first study that has compared cisgender and transgender androphilic males from the same high fertility population and demonstrated that the two do not differ with respect to the familial patterning of male androphilia.

## Introduction

Male androphilia refers to male sexual attraction and arousal to other adult males. The manner in which male androphilia is publically expressed varies cross-culturally, but generally takes one of two primary forms: cisgender and transgender [1–3]. *Cisgender male androphiles* behave in a relatively masculine manner, whereas *transgender male androphiles* typically behave in a relatively feminine manner. Both cisgender and transgender male androphiles can occur in the same culture, but typically one or the other tends to predominate [3]. Previous research has noted that the cisgender form of male androphilia is typically expressed in Euro-American cultures, whereas the transgender form tends to prevail in non-Euro-American cultures [1].

Despite significant differences in gender role enactment and identity, cross-cultural research suggests that both the transgender and cisgender forms of male androphilia share numerous biopsychological correlates [2]. For example, compared to males who are gynephilic (i.e., sexually attracted to adult females), both cisgender and transgender androphilic males occur at a similar population rates, are later born among their siblings, have more older brothers, come from larger families, have more androphilic male relatives, exhibit little or no reproductive output, recall elevated gender atypicality and separation anxiety in childhood, and exhibit a preference for female-typical occupations [2, 4]. These converging lines of evidence suggest that cisgender and transgender androphilic males are different expressions of the same underlying trait, and that both share common biological foundations [2, 5].

Regardless of how male androphilia is publically expressed, this trait represents an evolutionary puzzle when expressed to the exclusion of gynephilia because it hinders direct reproduction [6, 7]. Compounding this puzzle is the fact that both twin [8–11] and molecular genetic studies [12–14] indicate that male androphilia is influenced by genetic factors, and is thus, at least partially heritable. A more precise understanding of the nature of this heritability can be obtained by conducting family studies, which shed light on the way male androphilia clusters in families (i.e., whether they are grouped on the maternal line, paternal line, or both).

In Euro-American cultures, various family studies have consistently demonstrated that cisgender androphilic (gay) males have more androphilic male brothers than gynephilic males, thus bolstering the conclusion that male androphilia is familial [6, 15]. However, these studies have provided a mixture of results with respect to whether male androphilia is inherited through the maternal line, paternal line, or both. Some studies demonstrate that gay men have a preponderance of gay male relatives, but only in the maternal line [16–18]. These studies suggest that X-linkage factors might play an essential role in the expression of male androphilia because males can only share X-linked genes with maternal kin. At the same time, other studies demonstrate that gay men exhibit a preponderance of gay male relatives in both the maternal and the paternal lines [6, 15]. This pattern of familial clustering is consistent with the conclusion that genes for male androphilia can be inherited from both parents through autosomal-linked genes.

The discrepancies between the family studies described above may be partially explained by the low fertility rates typical in most Euro-American cultures [19]. Families in Euro-American societies tend to cease reproduction after obtaining a certain number of children, or after having one child of each sex. The existence of these so called “stopping rules” may obscure the familial patterning of low-frequency traits such as male androphilia [20], as has been shown to be the case for other well-established biodemographic correlates of male sexual orientation such as the fraternal birth order effect [21–23].

Additionally, because cisgender androphilic males in the West identify as men, and there are numerous cultural reasons for not disclosing one’s sexual orientation (e.g., [24, 25]) it is possible that Euro-American participants may report inaccurate or incomplete information regarding the sexual orientation of both themselves and their male relatives. This potential confound is circumvented in cultures in which androphilic males are recognized as occupying a third gender category that is distinct from men and women, as is the case in some non-Euro-American cultures. Identification by males as third gender in these non-Euro-American cultures is therefore a reliable indicator of male androphilia. As such, family studies conducted in high fertility non-Euro-American populations, where androphilic males are recognized as a third gender, are valuable complements to studies carried out with gay men in lower fertility Euro-American populations.

Research conducted in Samoa—a Polynesian, high fertility population—has consistently demonstrated that the families of transgender androphilic males (known locally as a third gender—*fa’afafine*) have a higher proportion of androphilic male relatives (i.e. brothers, uncles, and cousins) when compared to the families of gynephilic males [5, 26, 27]. These studies showed that *fa’afafine* have a preponderance of *fa’afafine* relatives in both their maternal and paternal lines, suggesting that autosomal-linkage factors are important for the inheritance of male androphilia. However, the rate of male androphilia among relatives with whom participants were more likely to share X-linked genes (i.e., maternal uncles and cousins via maternal aunts) was higher for *fa’afafine* than gynephilic males [5], furnishing some support, as well, for the role of X-linkage factors in the maintenance of male androphilia. Thus, evidence derived from family studies in Samoa indicates that male androphilia is familial, and is influenced by both autosomal and X-linkage factors.

Data from these family studies have also been used to estimate the population prevalence rate of male androphilia in Samoa. VanderLaan and colleagues [26] reported a population prevalence rate between 1.43–4.65%. In a larger follow-up study, Semenyna and colleagues [5] reported similar, but more circumscribed results (0.61–3.51%). These rates are comparable to those obtained for gay men in Euro-American cultures (~1–5%) (e.g., [20, 28]).

Research conducted in Samoa provides the first empirical evidence that transgender male androphilia clusters within families. However, further evidence from additional non-Euro-American, high fertility populations would help to elucidate the patterns of inheritance that typify transgender and cisgender male androphiles, especially if that evidence was derived from a culture that is unrelated to Samoa. In addition, such data could be used to generate prevalence rates of male androphilia in additional non-Euro-American populations, thereby addressing calls for such research [29]. With this in mind, we examined the familial patterning of male androphilia among the Istmo Zapotec of Oaxaca, Mexico.

The Zapotec are an indigenous Mesoamerican culture found primarily in the southern Mexican state of Oaxaca [30]. Zapotec civilization predates Spanish conquest by millennia, and despite foreign influences, Zapotec culture still remains an integral part of Oaxacan communities [31]. Federal statistics show that of the ~400,000 individuals in Mexico who speak Zapotec, 87% of them resided in Oaxaca [32]. Furthermore, fertility rates in Oaxaca are estimated to be higher than neighboring Mexican states [33], as well as a variety of Euro-American countries (e.g., Canada, Italy, US, and UK) [19].

The Istmo Zapotec—a subgroup of Zapotec living in the Istmo region of Oaxaca—recognize three genders: men, women, and *muxes*. The term *muxe* likely originates from a Zapotec adaptation of the Spanish word *mujer*, which means “woman” [34]. *Muxes* are androphilic males who commonly take the receptive role during anal intercourse. Qualitative studies indicate that *muxes* exhibit gender atypical behavior from an early age [34–36]. These observations are supported by recent quantitative research demonstrating that *muxes* recall elevated indicators of childhood separation anxiety [37], a trait most often displayed by females [38, 39].

Istmo Zapotec recognize two types of *muxes*: *muxe gunaa* and *muxe nguiiu* (i.e., Zapotec for *muxe* “woman” and *muxe* “man,” respectively). *Muxe gunaa* are transgender androphilic males who present publically in a relatively feminine manner, similar to the Samoan *fa’afafine*. *Muxe nguiiu* are cisgender androphilic males who present publically in a relatively masculine manner, similar to Euro-American gay men. Within the Istmo region of Oaxaca, both the transgender form of male androphilia (*muxe gunaa*) and the cisgender form (*muxe nguiiu*) occur at appreciable rates. Despite differences in gender expression, both types of *muxes* are relatively feminine when compared to their gynephilic male counterparts, as is generally the case for androphilic males worldwide (e.g., [40–44]). Interestingly, a widespread belief among the Istmo Zapotec is that *muxes* “run in families,” and their status as *muxe* is determined at birth by biological factors [34, 35].

Our study tested this folk belief by examining whether male androphilia is familial among the Istmo Zapotec. Given that substantial numbers of both transgender and cisgender *muxes* exist among the Istmo Zapotec, a unique *within*-culture comparison can be made on the proportion of androphilic male relatives in the families of both cisgender (*muxe nguiiu*) and transgender (*muxe gunaa*) androphilic males. Thus, the first aim of the present study was to compare the familial patterning and prevalence of androphilic male relatives between *muxe gunaa* and *muxe nguiiu*. Next, the prevalence of *muxe* relatives (i.e., brothers, uncles, and cousins) was compared between the families of Istmo Zapotec *muxes* (cisgender and transgender combined) and gynephilic males. In addition, we conducted within-group comparisons to determine whether there were any differences in the prevalence of androphilic male relatives between paternal and maternal kin categories (i.e., uncles, male cousins via uncles, male cousins via aunts, and all categories combined) for the probands of Istmo Zapotec men and *muxes*. Finally, a population prevalence rate of male androphilia among the Istmo Zapotec was calculated. Based on these aims, and on the literature mentioned above, our hypotheses and predictions were as follows:

**Hypothesis 1.** Both transgender and cisgender androphilic males have similar familial patterning of male androphilia.
**Prediction 1.** Istmo Zapotec *muxe gunaa* (transgender) and *muxe nguiiu* (cisgender) will not differ significantly with respect to the proportion of *muxe* relatives within their families.
**Hypothesis 2.** Androphilic males have more androphilic male relatives than gynephilic males.
**Prediction 2.** Istmo Zapotec *muxes* (both cisgender and transgender combined) will have significantly more *muxe* relatives than Istmo Zapotec gynephilic males.
**Hypothesis 3.** Androphilic males in non-Euro-American cultures have similar familial patterning of male androphilia in both maternal and paternal lines.
**Prediction 3.** Istmo Zapotec *muxes* will not differ significantly with respect to the prevalence of *muxe* relatives between the paternal and maternal kin categories (i.e., uncles, male cousins via uncles, and male cousins via aunts, and all combined).
**Hypothesis 4.** The prevalence rate of male androphilia is similar across cultures (~1–5%).
**Prediction 4.** The prevalence rate of *muxes* among the Istmo Zapotec will be similar to the prevalence rate of male androphilia found across cultures (~1–5%).

## Method

### Ethic statement

This research was approved by the University of Lethbridge Human Subjects Research Ethics Committee (Protocol #2015–069). Canadian, USA and French foreigner nationals are permitted to conduct research in Mexico for a period of 180 days if they have a valid passport [45]. All the authors held valid passports from these countries and our field trips did not exceed this period of time. The authors also confirmed with the Mexican Embassy in Ottawa, Canada, and the Mexican Consulate in Calgary, Canada, that a research permit from Mexican authorities was not required to conduct this study. While in Juchitán, we met with some of the leaders of the *muxe* community to explain our research and these leaders offered their support. Furthermore, we visited the local police station and the police were made aware of our research activities. As such, this research was conducted in compliance with local research regulations in Mexico.

### Participants

Consistent with previous family studies conducted in Samoa [5, 26], all participants were recruited using a network sampling procedure which consisted of contacting randomly chosen initial participants, who gave referrals for additional participants, who in turn provided further referrals, and so on. Data were collected in the city of Juchitán de Zaragoza, as well as 14 other towns and villages within the Juchitán and Tehuantepec districts in the Istmo region of Oaxaca, Mexico. Three separate field trips took place between November-December, 2015, February-March 2016, and November-December 2016. Participants were required to provide informed written consent prior to participating in the study.

Participants were asked to report their gender as either men or *muxe*. If they identified as *muxes*, they were then asked to identify as either *muxe gunaa* or *muxe nguiiu*. A total of 171 gynephilic men and 169 *muxes* (110 *muxe gunaa* and 59 *muxe nguiiu*) were interviewed for this study. Participants could answer the questionnaires alone, but it was not unusual for them to also receive assistance from relatives if they were nearby. None of the participants were brothers or first cousins. Participants’ sexual orientations were assessed using a 7-point Kinsey scale [46], which asked about sexual feelings over the previous year. Participants’ response options ranged from *Sexual feelings only toward females* (i.e., exclusively gynephilic; Kinsey rating = 0) to *Sexual feelings only toward males* (i.e., exclusively androphilic; Kinsey rating = 6). All men identified as exclusively (Kinsey rating = 0, *n* = 165 men) or predominantly gynephilic (Kinsey rating = 1, *n* = 6 men). All *muxes* identified as predominantly (Kinsey rating = 5, *n* = 7 *muxe nguiiu*) or exclusively androphilic (Kinsey rating = 6, *n* = 52 *muxe nguiiu*; *n* = 110 *muxe gunaa*).

### Biographic information

Participants were asked to report information regarding their age (in years). A one-way analysis of variance (ANOVA) showed that the average age of gynephilic men (*M* = 30.33, *SD* = 9.18), *muxe gunaa* (*M* = 30.58, *SD* = 9.15), and *muxe nguiiu* (*M* = 31.37, *SD* = 10.08), did not differed significantly (*F*[2, 337] = .275, *p* = .760). Participants were also asked to report their average weekly income with a scale that ranged from 1 (0–250 Mexican Pesos) to 9 (more than 2000 Mexican Pesos). A one-way ANOVA revealed that the average level of income for gynephilic men (*M* = 5.00, *SD* = 2.47), *muxe gunaa* (*M* = 4.72, *SD* = 2.24), and *muxe nguiiu* (*M* = 5.24, *SD* = 2.58), did not differed significantly (*F*[2, 337] = .951, *p* = .387). As such, none of the biographic variables were used as covariates when conducting inferential statistics.

### Measures

Participants were interviewed using questionnaires, which were available in Spanish after being translated and back-translated by two fluent Spanish-English speakers. Two of the authors (FRG, LC) and Spanish-speaking research assistants were available to answer participants’ questions. When participants were not fully fluent in Spanish, a Zapotec-speaking research assistant was also present for interviews. Questions were read out loud by research assistants in Spanish or Zapotec as necessary.

Participants reported the total number of biological brothers they had, as well as all biological male relatives (i.e., uncles, male cousins via aunts, and male cousins via uncles) for both the paternal and maternal sides of their families. An additional category was created combining maternal uncles and male cousins via aunts in order to test for potential X-linkage factors of male androphilia. These kin categories are the only males with whom probands might share common X-linked genes. Brothers were not included in this category because they share both X-linked genes and the same Y chromosome as probands, thus confounding comparisons between the paternal and maternal lines. The participants identified how many of those relatives were *muxes*. This information was used to calculate the proportion of *muxes* relatives within each kin category for each participant. These proportions were then averaged for each kin category within each group so as to have a mean proportion of *muxe* relative to conduct group comparisons.

Some of the participants had relatives who moved outside of the Istmo to different states within Mexico or to different countries that are known to have lower fertility rates (e.g., Mexico City, United States). Since our primary aim in this study was to analyze the prevalence of male androphilia within the Istmo region of Oaxaca, only male relatives whose parents had spent their entire reproductive history within the Istmo were included in the analysis.

### Statistical analyses

Mann-Whitney *U* tests where employed when comparing the average proportion of *muxe* relatives between Istmo Zapotec *muxe gunaa* and *muxe nguiiu* in the paternal line, maternal line, and both lines combined (Table 1). Within group comparisons were conducted comparing the paternal and maternal relatives of *muxe gunaa* and *muxe nguiiu* using Wilcoxon signed-rank tests (Table 2). Finally, additional within group comparisons were conducted using Friedman tests in order to compare the prevalence of maternal and paternal *muxe* relatives across different kin categories (i.e., uncles, male cousins via uncles, and male cousins via aunts) for both *muxe gunaa* and *muxe nguiiu* (Table 3). Post hoc analyses for the Friedman tests were conducted using Wilcoxon signed-rank tests (Table 3). The same between-group and within-group statistical analyses were used when comparing Istmo Zapotec *muxes* (both cisgender and transgender combined) to gynephilic men (Tables 4–6). Given the numerous statistical comparisons carried out, a more conservative critical alpha of 0.01 was used in order to control the type I error rate. Due to the skewed nature of the data, all Cohen’s *d* effect sizes should be interpreted with caution. For Tables 2, 3, 5 and 6, the *r* effect sizes were calculated using the *z* scores from the Wilcoxon signed-rank test. Effect size interpretations are as follows: *r* = .1 small, .3 medium, .5 large; *d* = .2 small, .5 medium, and .8 large [47, 48].

**Table 1:** Comparisons of the prevalence of muxe relatives among muxe gunaa and muxe nguiiu.

	*Muxe Gunaa*					*Muxe Nguiiu*					Mann-Whitney *U*	*p*	Cohen’s *d*
	*n*	*M*	*SD*	Number of *Muxe* Relatives/ Total Number of Male Relatives	Percentage (%) of *Muxe* Relatives	*n*	*M*	*SD*	Number of *Muxe* Relatives/ Total Number of Male Relatives	Percentage (%) of *Muxe* Relatives			
Paternal and maternal relatives	110	.061	.068	138/2571	5.37	59	.053	.063	59/1145	5.15	3459.5	.469	.12
Paternal relatives:	104	.058	.101	58/1087	5.34	56	.059	.090	30/546	5.49	2891.5	.933	.00
Uncles	98	.067	.182	18/283	6.36	55	.057	.199	7/162	4.32	2857.5	.320	.05
Male cousins via uncles	78	.032	.086	15/378	3.97	46	.082	.189	15/209	7.17	1602	.156	-.37
Male cousins via aunts	83	.077	.189	25/426	5.87	41	.035	.105	8/175	4.57	1902	.129	.25
Maternal relatives:	105	.074	.146	68/1242	5.47	56	.059	.112	25/499	5.01	3179	.335	.11
Uncles	96	.053	.162	13/307	4.23	55	.059	.174	8/162	4.94	2591.5	.750	-.04
Male cousins via uncles	79	.032	.086	16/422	3.79	45	.067	.191	13/178	7.30	1765.5	.922	-.26
Male cousins via aunts	88	.103	.215	39/513	7.60	40	.029	.100	4/159	2.52	2173	.006	.40
Uncles and male cousins via aunts	104	.078	.159	52/820	6.34	56	.052	.148	12/321	3.74	3273	.114	.17
Brothers	91	.051	.167	12/242	4.96	47	.046	.173	4/100	4.00	2224.5	.487	.03

**Table 2:** Within group comparisons of the prevalence of paternal and maternal muxe relatives of muxe gunaa and muxe nguiiu.

		Paternal		Maternal		Wilcoxon signed-rank (*z*)	*p*	Effect Size *r = z*/(*n*)^1/2^	Effect Size Cohen’s *d*
	*n*	*M*	*SD*	*M*	*SD*				
*Muxe gunaa*	99	.059	.101	.074	.146	.459	.646	.05	-.12
Uncles	84	.067	.182	.053	.162	.972	.331	.11	.08
Male cousins via uncles	56	.032	.086	.032	.086	.751	.453	.06	.00
Male cousins via aunts	69	.077	.189	.103	.215	1.12	.264	.13	-.13
*Muxe nguiiu*	53	.059	.090	.059	.112	.299	.765	.04	.00
Uncles	51	.057	.199	.059	.174	.105	.916	.01	-.01
Male cousins via uncles	39	.082	.189	.067	.191	.035	.972	.01	.08
Male cousins via aunts	28	.035	.105	.029	.100	.339	.735	.06	.06

**Table 3:** Comparison of the prevalence of muxe relatives across kin categories within muxe gunaa and muxe nguiiu participants for the paternal line, maternal line, and both lines combined.

	*n*	Uncles		Male Cousins via Uncles		Male Cousins via Aunts		Friedman Test *χ*2 (*df* = 2)	*p*	Effect Size *r = z*/(*n*)^1/2^	Effect Size Cohen’s *d*
		*M*	*SD*	*M*	*SD*	*M*	*SD*				
*Muxe gunaa*											
Paternal and maternal	95	.065	.154	.034	.068	.088	.169	7.62	.022	.13, .08, .27^a^	.26, -.14, -.42^a^
Paternal	66	.067	.182	.032	.086	.077	.189	1.92	.383	.10, .02, .15	.25, -.05, -.31
Maternal	71	.053	.162	.032	.086	.103	.215	6.69	.035	.06,.20, .22	.16, -.26, -.43
*Muxe nguiiu*											
Paternal and maternal	48	.056	.119	.069	.118	.035	.093	1.13	.569	.14, .16, .18	-.11, .20, .32
Paternal	35	.057	.199	.082	.189	.035	.105	.950	.622	.21, .11, .09	-.13, .14, .31
Maternal	35	.059	.174	.067	.191	.029	.100	.216	.898	.02, .10, .12	-.04, .21, .25

All effect size estimates are listed in order of comparing uncles to male cousins via uncles; uncles to male cousins via aunts; male cousins via uncles to male cousins via aunts.

^a^ Post-hoc Wilcoxon’s test between overall male cousins via uncles versus overall male cousins via aunts was significant (*p* = .008). However, the preceding omnibus test was not.

**Table 4:** Comparisons of the prevalence of muxe relatives among muxes versus gynephilic male participants.

	*Muxes*					Gynephilic Males					Mann-Whitney *U*	*p*	Cohen’s *d*
	*n*	*M*	*SD*	Number of *Muxe* Relatives/ Total Number of Male Relatives	Percentage (%) of *Muxe* Relatives	*n*	*M*	*SD*	Number of *Muxe* Relatives/ Total Number of Male Relatives	Percentage (%) of *Muxe* Relatives			
Paternal and maternal relatives	169	.058	.066	197/3716	5.30	171	.039	.058	129/3183	4.05	17388	.001	.31
Paternal relatives:	160	.059	.097	88/1633	5.39	163	.032	.068	63/1429	4.41	15127.5	.002	.32
Uncles	153	.063	.188	25/445	5.62	153	.032	.123	15/427	3.51	12472.5	.076	.20
Male cousins via uncles	124	.050	.135	30/587	5.11	117	.047	.146	26/528	4.92	7620.5	.305	.02
Male cousins via aunts	124	.063	.167	33/601	5.49	116	.045	.149	22/474	4.64	7597	.255	.11
Maternal relatives:	161	.068	.135	93/1741	5.34	165	.050	.103	63/1495	4.21	14394.5	.124	.15
Uncles	151	.055	.166	21/469	4.48	155	.033	.133	13/449	2.90	12285	.162	.15
Male cousins via uncles	124	.045	.134	29/600	4.83	131	.044	.134	22/532	4.14	8222	.786	.01
Male cousins via aunts	128	.080	.189	43/672	6.40	127	.065	.158	28/514	5.45	8587.5	.291	.09
Uncles and male cousins via aunts	160	.069	.155	64/1141	5.61	164	.049	.106	41/963	4.26	14009	.176	.15
Brothers	138	.050	.168	16/342	4.68	129	.006	.053	3/259	1.16	9789	.001	.35

**Table 5:** Comparisons of the prevalence of muxe relatives in the paternal and maternal lines of muxes and gynephilic participants.

	*n*	Paternal		Maternal		Wilcoxon signed-rank (*z*)	*p*	Effect Size *r = z*/(*n*)^1/2^	Effect Size Cohen’s *d*
		*M*	*SD*	*M*	*SD*				
*Muxes*	152	.059	.097	.068	.135	.176	.860	.01	-.08
Uncles	135	.063	.188	.055	.166	.519	.604	.04	.05
Male cousins via uncles	95	.050	.135	.045	.134	.389	.697	.04	.04
Male cousins via aunts	97	.063	.167	.080	.189	.908	.364	.09	-.10
Gynephilic males	157	.032	.068	.050	.103	1.76	.079	.14	-.21
Uncles	137	.032	.123	.033	.133	.315	.753	.03	-.01
Male cousins via uncles	89	.047	.146	.044	.134	.037	.970	.00	.02
Male cousins via aunts	86	.045	.149	.065	.158	1.44	.149	.16	-.13

**Table 6:** Comparison of the prevalence of muxe relatives across kin categories within muxes and gynephilic male participants for the paternal line, maternal line, and both lines combined.

	*n*	Uncles		Male Cousins via Uncles		Male Cousins via Aunts		Friedman Test *χ*2 (*df* = 2)	*p*	Effect Size *r = z*/(*n*)^1/2^	Effect Size Cohen’s *d*
		*M*	*SD*	*M*	*SD*	*M*	*SD*				
*Muxes*											
Paternal and maternal	142	.062	.143	.046	.089	.070	.150	3.03	.220	.04, .00, .12	.13, -.05, -.19
Paternal	101	.063	.188	.050	.135	.063	.167	1.94	.379	.03, .05, .08	.07, .00, -.08
Maternal	106	.055	.166	.045	.134	.080	.189	4.01	.134	.03, .10, .13	.07, -.14, -.21
Gynephilic males											
Paternal and maternal	147	.030	.076	.049	.129	.051	.106	2.71	.258	.12, .13, .04	-.18, -.23, -.02
Paternal	90	.032	.123	.047	.146	.045	.149	.747	.688	.07, .05, .05	-.11, -.10, -.01
Maternal	103	.033	.133	.044	.134	.065	.158	8.09	.018	.15, .18, .13	-.08, -.22, -.14

All follow-up pairwise comparisons were conducted using Wilcoxon’s test with no tests reaching significance (all *p* ≥ .055). All effect size estimates are listed in order of comparing uncles to male cousins via uncles; uncles to male cousins via aunts; male cousins via uncles to male cousins via aunts.

## Results

### Comparison between muxe gunaa and muxe nguiiu

Consistent with Prediction 1, the two types of *muxes* did not significantly differ with respect to the proportion of *muxe* relatives overall (i.e., maternal and paternal lines combined) (Table 1). Additionally, *muxe gunaa* and *muxe nguiiu* did not differ significantly with respect to the prevalence of *muxe* relatives in either their combined paternal or combined maternal lines. Within the maternal line, *muxe gunaa* were found to have significantly more *muxe* cousins via aunts compared to *muxe nguiiu*. The prevalence of *muxe* relatives in the category “X-chromosome-linked male kin” (i.e., maternal uncles and male cousins via maternal aunts combined) did not differ significantly between groups. Lastly, no significant difference was observed when comparing the proportion of *muxe* brothers between *muxe gunaa* and *muxe nguiiu* probands.

For both types of *muxes*, no significant differences were observed within groups for the prevalence of androphilic male relatives in paternal and maternal kin categories (i.e., uncles, male cousins via uncles, and male cousins via aunts) (Table 2). When comparing the prevalence of *muxe* relatives among uncles, cousins via uncles, and cousins via aunts (Table 3), no significant differences were found for *muxe gunaa*. Similarly, *muxe nguiiu* showed no significant differences in the proportion of *muxe* relatives in any of these kin categories.

### Comparison between muxes and gynephilic males

Given that proportions of *muxe* relatives among the families of *muxe gunaa* and *muxe nguiiu* were largely equivalent, the two *muxe* types were combined in order to compare them to gynephilic males. Consistent with Prediction 2, *muxe* probands had significantly more *muxe* relatives overall (i.e., maternal and paternal lines combined) than gynephilic male probands (Table 4). *Muxe* probands also had a significantly higher proportion of androphilic male paternal relatives compared to gynephilic males, whereas maternal relatives did not differ significantly between the groups. Within *muxes*’ paternal line, no individual kin category was found to be driving the preponderances of paternal *muxe* relatives compared to those of gynephilic males. When considering the category “X-chromosome-linked male kin” (i.e., maternal uncles and male cousins via maternal aunts combined), no significant differences in the prevalence of *muxe* relatives were found between groups. Lastly, *muxes* reported having significantly more *muxe* brothers than gynephilic males.

Consistent with Prediction 3, no significant differences were observed for the prevalence of androphilic male relatives in paternal and maternal kin categories (i.e., uncles, male cousins via uncles, and male cousins via aunts) within the families of *muxes* (Table 5). The same was also true for families of gynephilic males. Finally, when comparing the prevalence of *muxe* relatives among uncles, male cousins via uncles, and male cousins via aunts (Table 6), both *muxes* and gynephilic men showed no significant differences.

### Population prevalence estimate of male androphilia among the Istmo Zapotec

The data collected in the current study were used to calculate a population prevalence estimate of *muxes* (i.e., male androphilia) among the Istmo Zapotec. Consistent with previous studies [5, 26], the population prevalence estimate was comprised of the overall proportion of *muxe* relatives (i.e., paternal and maternal lines combined, including brothers) in relation to all male relatives (listed in Table 4). Specifically, the upper bound of the population prevalence estimate was calculated using the proportion of *muxe* relatives among *muxe* probands, while the lower bound was calculated using the proportion of *muxe* relatives among gynephilic male probands. Given the binomial nature of these estimates (i.e., relatives either are *muxes*, or are not), the *SD* was calculated as $\sqrt{npq}$, where *n* is the total number of male relatives (i.e., 3716 for *muxes* and 3183 for gynephilic men), *p* is the proportion of male relatives who are *muxes* (i.e., 197/3716 for *muxes* and 129/3183 for gynephilic men), and *q* is the proportion of male relatives who are not *muxes* (i.e., 1 –*p*). The standard deviations (*SD*s) of these estimates were used to calculate 95% confidence intervals on the upper bound (i.e., the prevalence of *muxe* relatives among *muxe* probands) and the lower bound (i.e., the prevalence of *muxe* relatives among gynephilic male probands) respectively.

For the *muxe* probands, a frequency of 197 *muxe* relatives out of 3716 total male relatives (5.30%) yielded a *SD* of 13.66, which represents 0.37% of the total number of *muxe* probands’ male relatives. For the gynephilic male probands, a frequency of 129 *muxe* relatives out of 3183 total male relatives (4.05%) yielded a *SD* of 11.13, which represents 0.35% of all gynephilic male probands’ relatives. The 95% confidence intervals (CIs) for the prevalence of *muxe* relatives were calculated as $p\pm 1.96\;\frac{SD}{n}$. Similar to the *SD* formula, *p* is the proportion of male relatives who are *muxes* and *n* is the total number of male relatives. Therefore, the 95% CI for the prevalence rate of *muxe* relatives was 4.58–6.02% (0.0458, 0.0602) for *muxe* probands, and 3.37–4.74% (0.0337, 0.0474) for gynephilic male probands. Given previous research suggesting that androphilic males have more androphilic male relatives than gynephilic males [5, 26, 27], and that the CI for the *muxe* probands was higher than the CI of gynephilic male probands, we used the upper bound of the CI of the *muxe* probands (6.02%) and the lower bound of the CI of the gynephilic male probands (3.37%), as the upper and lower bound for the population prevalence rate of male androphilia, respectively. As such, we estimate that the true rate of androphilia among the Istmo Zapotec must falls between 3.37–6.02%, thus providing some support for Prediction 4.

## Discussion

In order to determine whether male androphilia clusters within families among the Istmo Zapotec, the current study compared the proportion of *muxe* relatives in the paternal and maternal lines of gynephilic males and *muxe*s. Comparisons between transgender (*muxe gunaa*) and cisgender (*muxe nguiiu*) *muxes* revealed that both reported analogous family patterning of male androphilia. This held true when comparing the paternal and maternal lines separately, and when combined. There was, however, one significant difference observed between the two types of *muxes*. *Muxe gunaa* reported having more androphilic male cousins via maternal aunts than did *muxe nguiiu* (Table 1). Given that a substantial body of research demonstrates that transgender and cisgender male androphiles share numerous biodemographic correlates [2], there is no *a priori* reason to predict why this pattern would emerge within this specific kin category alone. This difference, while statistically significant, is probably an artifact of the small sample size for *muxe nguiiu* in this kin category (*n* = 40). As such it is likely to be the result of type I error. These subtle differences did not overshadow the larger pattern, which showed that *muxe gunaa* and *muxe nguiiu* did not differ with respect to the clustering of male androphilia within their families.

After establishing that the two types of *muxes* had comparable proportions of androphilic male relatives, groups were combined in order to compare them to gynephilic males. Consistent with previous family studies conducted in both Euro-American and non-Euro-American cultures, the results presented here provide evidence that Istmo Zapotec *muxes* have more *muxe* relatives than gynephilic males. *Muxes* reported having more *muxe* relatives in the paternal line than did gynephilic males (Table 4). However, when comparing within groups, there were no significant differences with respect to the prevalence of *muxe* relatives in the paternal and maternal lines for both *muxe* and gynephilic male probands (Table 5). Taken together, the results suggest that male androphilia clusters in the families of Istmo Zapotec *muxes*, and this clustering is equivalent in both the maternal and paternal lines.

It has been suggested that male androphilia is not a trait governed by simple Mendelian inheritance (i.e., single gene accounting for the expression of a trait), but requires instead a multifactorial genetic explanation involving both X-linkage as well as autosomal-linkage factors [13, 14, 49]. The current study provides findings that are consistent with this conclusion among the Istmo Zapotec. We did not find strong evidence implicating X-linked genetic factors as *exclusively* underpinning male androphilia because *muxe* probands did not exhibit a significant preponderance of *muxe* relatives in their maternal lines overall (Table 5), nor among the specific kin with whom they are capable of sharing X-linked genes (i.e., maternal uncles and cousins via maternal aunts) (Table 4). The fact that our data did not support an exclusively X-linked genetic explanation for male androphilia does not mean that genes on the X-chromosome do not play a role in the maintenance of male androphilia within this culture. Instead, it is likely that Istmo Zapotec *muxes* and androphilic males elsewhere inherit both autosomal and sex-linked genes that act in synchrony (i.e., polygenic inheritance) to influence the development and expression of sexual orientation. In supporting this argument, both X-linked (i.e., Xq28) and autosomal (i.e., the centromeric region of the chromosome 8) genetic regions appear to be involved in the development of male androphilia [12–14].

In addition to examining familial patterning of male androphilia, this study also produced a population prevalence estimate of male androphilia among the Istmo Zapotec. The upper and lower bounds for this estimate were the proportion of *muxe* relatives among the families of all *muxes* combined and gynephilic males, respectively (Table 4). As such, the true prevalence of male androphilia among the Istmo Zapotec is estimated to fall between 3.37–6.02%. This is largely consistent with estimates derived from Euro-American cultures, where the population prevalence of cisgender “gay men” falls between ~1–5% [20, 28]. The current estimate, while valuable, does not tell us the actual differences in prevalence between cisgender and transgender *muxes* in the Istmo, as participants were not asked to identify their *muxe* relatives as being *muxe nguiiu* or *muxe gunaa*. Nonetheless, the population prevalence rate of *muxes*, which is composed by a highly noticeable number of *muxe gunaa*, appears to be much higher than the prevalence of Euro-American transsexual women (i.e., biological males who opt for sex reassignment surgery), which is notably smaller (i.e.,< 0.001%) [50–52].

The Istmo Zapotec are somewhat unique in that both cisgender and transgender forms of male androphilia occur at appreciable rates in the culture. It is unclear, however, how androphilic males within the same culture come to adopt either a cisgender or transgender identity. Semenyna and colleagues [5] argued that the differences in gender identity and gender-role enactment between cisgender and transgender androphilic males are a result of the manner in which male androphilia is culturally elaborated. There are several factors that could influence whether an androphilic male in the Istmo Zapotec will adopt a cisgender instead of a transgender identity. Primary among them are variations in female-typical behavior, acceptance/tolerance of feminine gender expression in males by family members or peers, and exposure to Euro-American culture. The Istmo Zapotec represent a suitable model in which to test whether these or other factors are responsible for the gender role enactment of the different *muxe* types, and what specific influences canalize the development of either a transgender or a cisgender identity among androphilic males.

This study, coupled with other family and twin studies (see above), indicates that male androphilia is familial, while molecular genetic studies indicate that it is partly influenced by genetic factors. These insights, however, raises further questions as to how exactly genes associated with male androphilia persist across generations given that androphilic males reproduce at far lower rates than gynephilic males, if at all [2, 6]. The two most prominent explanations for this evolutionary conundrum are the Kin Selection Hypothesis (KSH), and the Sexual Antagonistic Gene Hypothesis (SAGH) [29].

The KSH holds that genes for male androphilia persist over evolutionary time if androphilic males behave altruistically (e.g., provide care or resources) toward their close kin with whom they share numerous copies of their genes by virtue of common descent. This altruism may then increase kin fitness, thus offsetting the costs of not reproducing directly [53]. Research conducted on cisgender androphilic males in industrialized cultures has provided little support for the KSH [2, 54–58]. In contrast, research conducted on transgender androphilic males in Samoa has repeatedly found support for the KSH by means of elevated kin directed altruism among *fa’afafine* [59–64]. Because the transgender form of male androphilia appears to be ancestral to the cisgender form [65], the former likely represents a better model when testing evolutionary hypotheses pertaining to male androphilia than the later.

The SAGH––a complementary rather than competing hypothesis––states that genes associated with male androphilia reduce reproduction when present in males, but increase reproduction when present in the female relatives of androphilic males [16]. Some studies conducted on cisgender androphilic male in Euro-American cultures have provided results consistent with the SAGH (Italy: [16, 66, 67]; Caucasian participants in the UK: [18]), whereas others have not (USA: [6, 68]; non-Caucasian participants in the UK: [18]). It is possible that the existence of reproductive stopping rules, which leads to lower fertility rates in Euro-American cultures, limits the increase in female reproduction that is hypothesized by the SAGH. However, studies of the SAGH in Samoa have shown that while the maternal grandmothers and mothers of *fa’afafine* demonstrate elevated reproduction, maternal aunts do not, leaving support for the SAGH equivocal at present [2, 69].

In line with the KSH, Gómez and colleagues [37] demonstrated that *muxes* recall elevated indicators of childhood separation anxiety, which appears to be a developmental precursor to elevated kin-directed altruism [59, 70]. Additionally, the results presented in this study are consistent with the SAGH, in that families of *muxes* were comprised of a higher number of total relatives compared to those of gynephilic males (Table 4). Nonetheless, a detailed comparison of the expression of kin-directed altruism, as well as the offspring production among the extended relatives of Istmo Zapotec gynephilic males and *muxes*, should be conducted in order to adequately test both the KSH and the SAGH. Given the inconsistencies across studies associated with the KSH and the SAGH, the Istmo Zapotec offers a compelling locale to conduct further tests among a non-Euro-American, high fertility population where male androphilia is commonly expressed in both the transgender and cisgender form.

### Limitations

There are several limitations in the current study that deserve comment. First, the identity status of *muxe* relatives was not corroborated with the male relatives themselves. That being said, none of the family studies that have been conducted to date have independently corroborated the sexual orientation of the relatives of participants. We suspect that Istmo Zapotec participants are probably less likely to misreport the sexual orientation of their male relatives compared to Euro-American study participants, because the former live in a culture where androphilic males constitute a distinct gender category, in which identification as *muxe*––whether *nguiiu* or *gunaa*––is both obvious and an unambiguous indicator of male androphilia [34, 35], whereas the latter do not. Furthermore, during many of the interviews, participants consulted with nearby members of their family in order to provide a precise report of their family pedigree. To a large extent, this reflects the reality of conducting fieldwork in a collectivistic cultural context where individuals are in close proximity to their family much of the time. The advantage of this is that information provided by the probands can be corroborated, corrected, or elaborated upon by those family members who are present. Moreover, the sexual activity and orientation of individuals is the source of much monitoring and gossip and, as such, is rarely kept secret to the extent that is possible in more individualist cultures. The disadvantage is that group differences could conceivably exist between those who provide information versus those who have input from family members. We did not perceive any differences in this regard, but we have no data that speaks to this possibility. This issue could be addressed in future studies.

Second, the aims of this study were to determine patterns of familial clustering and prevalence of male androphilia among the Istmo Zapotec as opposed to patterns and prevalence of the specific form of male androphilia (i.e., cisgender or transgender). Consequently, participants were not asked if their *muxe* relatives identified as *muxe gunaa* or *muxe nguiiu*. As such, we are only able to draw firm conclusions regarding the familial patterning of male androphilia in general, but not the specific ways cisgender and transgender male androphilia cluster in families.

Because male androphilia occurs at a relatively low frequency in any population, this study utilized a network sampling procedure. It is possible that this method produced a sampling bias, resulting in an unrepresentative sample of Istmo Zapotec *muxes*, men, or both. Efforts were made to avoid such bias by interviewing participants throughout the city of Juchitán de Zaragoza—the largest urban center in the Istmo region––as well as 14 towns and villages throughout the Juchitán and Tehuantepec districts in the Istmo region of Oaxaca. Nonetheless, future research conducted in the Istmo Zapotec could consider using random sampling procedures.

## Conclusion

This study on the Istmo Zapotec *muxes*, coupled with the research conducted on the Samoan *fa’afafine* [5, 26, 27] and Euro-American gay men [6, 15–18], suggests that having more androphilic male relatives is a cross-culturally universal aspect of male androphilia. This is the first study that has compared cisgender and transgender androphilic males in the same culture, showing that both report analogous proportions of androphilic male relatives, and a familial patterning of male androphilia that is overwhelmingly similar. The findings presented in this study are in accordance with previous research, which suggest that both forms of male androphilia share similar biological foundation. Future studies should directly assess different biological traits (e.g., genetic, morphological, and neurological) in order to determine the extent to which biological similarities between cisgender and transgender androphilic males exist.

## Supporting information

S1 Appendix Question about your father’s and mother’s side of the family (English Version). (DOCX)

S2 Appendix Question about your father’s and mother’s side of the family (Spanish Version). (DOCX)

## References

[1] Murray SO. Homosexualities. Chicago: The University of Chicago Press; 2000.

[2] Vasey PL, VanderLaan DP. Evolving research on the evolution of male androphilia. The Canadian Journal of Human Sexuality. 2014;23(3):137–47.

[3] Whitam FL, Mathy RM. Male homosexuality in four societies: Brazil, Guatemala, the Philippines, and the United States. New York: Praeger; 1986.

[4] Semenyna SW, Vasey PL. The relationship between adult occupational preferences and childhood gender nonconformity among Samoan women, men, and *fa'afafine*. Human Nature. 2016;27(3):283–95. doi: 10.1007/s12110-016-9258-7 27100108 10.1007/s12110-016-9258-7

[5] Semenyna SW, VanderLaan DP, Petterson LJ, Vasey PL. Familial patterning and prevalence of male androphilia in Samoa. The Journal of Sex Research. 2016;54(8):1077–84. doi: 10.1080/00224499.2016.1218416 27593894 10.1080/00224499.2016.1218416

[6] Schwartz G, Kim RM, Kolundzija AB, Rieger G, Sanders AR. Biodemographic and physical correlates of sexual orientation in men. Archives of Sexual Behavior. 2010;39(1):93–109. doi: 10.1007/s10508-009-9499-1 19387815 10.1007/s10508-009-9499-1

[7] Vasey PL, Parker JL, VanderLaan DP. Comparative reproductive output of androphilic and gynephilic males in Samoa. Archives of Sexual Behavior. 2014;43(2):363–7. doi: 10.1007/s10508-013-0195-9 24132776 10.1007/s10508-013-0195-9

[8] Alanko K, Santtila P, Harlaar N, Witting K, Varjonen M, Jern P, et al Common genetic effects of gender atypical behavior in childhood and sexual orientation in adulthood: A study of Finnish twins. Archives of Sexual Behavior. 2010;39(1):81–92. doi: 10.1007/s10508-008-9457-3 19172387 10.1007/s10508-008-9457-3

[9] Bailey JM, Dunne MP, Martin NG. Genetic and environmental influences on sexual orientation and its correlates in an Australian twin sample. Journal of Personality and Social Psychology. 2000;78(3):524–36 10743878 10.1037//0022-3514.78.3.524

[10] Kendler KS, Thornton LM, Gilman SE, Kessler RC. Sexual orientation in a US national sample of twin and non-twin sibling pairs. American Journal of Psychiatry. 2000;157(11):1843–6. doi: 10.1176/appi.ajp.157.11.1843 11058483 10.1176/appi.ajp.157.11.1843

[11] Långström N, Rahman Q, Carlström E, Lichtenstein P. Genetic and environmental effects on same-sex sexual behavior: A population study of twins in Sweden. Archives of Sexual Behavior. 2010;39(1):75–80. doi: 10.1007/s10508-008-9386-1 18536986 10.1007/s10508-008-9386-1

[12] Hamer DH. Genetics of sexual behavior In: Benjamin J, Ebstein RP, Belmaker RH, editors. Molecular genetics and the human personality. Washington, DC: American Psychiatric Publishing; 2002:257–72.

[13] Mustanski BS, DuPree MG, Nievergelt CM, Bocklandt S, Schork NJ, Hamer DH. A genomewide scan of male sexual orientation. Human Genetics. 2005;116(4):272–8. doi: 10.1007/s00439-004-1241-4 15645181 10.1007/s00439-004-1241-4

[14] Sanders AR, Martin ER, Beecham GW, Guo S, Dawood K, Rieger G, et al Genome-wide scan demonstrates significant linkage for male sexual orientation. Psychological Medicine. 2015;45(7):1379–88. doi: 10.1017/S0033291714002451 25399360 10.1017/S0033291714002451

[15] Bailey JM, Pillard RC, Dawood K, Miller MB, Farrer LA, Trivedi S, et al A family history study of male sexual orientation using three independent samples. Behavior Genetics. 1999;29(2):79–86. 10405456 10.1023/a:1021652204405

[16] Camperio Ciani A, Corna F, Capiluppi C. Evidence for maternally inherited factors favoring male homosexuality and promoting female fecundity. Proceedings of the Royal Society of London B: Biological Sciences. 2004;271(1554):2217–21. 10.1098/rspb.2004.2872 PMC1691850 15539346

[17] Hamer DH, Hu S, Magnuson VL, Hu N, Pattatucci AML. A linkage between DNA markers on the X chromosome and male sexual orientation. Science. 1993;261(5119):321–7. 8332896 10.1126/science.8332896

[18] Rahman Q, Collins A, Morrison M, Orrells JC, Cadinouche K, Greenfield S, et al Maternal inheritance and familial fecundity factors in male homosexuality. Archives of Sexual behavior. 2008;37(6):962–9. doi: 10.1007/s10508-007-9191-2 17665299 10.1007/s10508-007-9191-2

[19] Central Intelligence Agency. The world factbook: Country comparison: Birth rate. 2016. Available from: https://www.cia.gov/library/publications/the-world-factbook/rankorder/2127rank.html

[20] Gates GJ. How many people are lesbian, gay, bisexual, and transgender? The William Institute 2011 Available from: https://escholarship.org/uc/item/09h684x2

[21] Blanchard R, Lippa RA. Birth order, sibling sex ratio, handedness, and sexual orientation of male and female participants in a BBC Internet research project. Archives of Sexual Behavior. 2007;36(2):163–76. doi: 10.1007/s10508-006-9159-7 17345165 10.1007/s10508-006-9159-7

[22] Xu Y, Zheng Y. Fraternal birth order, handedness, and sexual orientation in a Chinese population. The Journal of Sex Research. 2017;54:10–18. doi: 10.1080/00224499.2015.1104530 26689787 10.1080/00224499.2015.1104530

[23] Zucker KJ, Blanchard R, Kim TS, Pae CU, Lee C. Birth order and sibling sex ratio in homosexual transsexual South Korean men: Effects of the male‐preference stopping rule. Psychiatry and Clinical Neurosciences. 2007;61(5):529–33. doi: 10.1111/j.1440-1819.2007.01703.x 17875032 10.1111/j.1440-1819.2007.01703.x

[24] D'Augelli AR, Grossman AH. Disclosure of sexual orientation, victimization, and mental health among lesbian, gay, and bisexual older adults. Journal of Interpersonal Violence. 2001;16(10):1008–27.

[25] D'Augelli AR, Hershberger SL, Pilkington NW. Lesbian, gay, and bisexual youth and their families: disclosure of sexual orientation and its consequences. American Journal of Orthopsychiatry. 1998;68(3):361–71. 9686289 10.1037/h0080345

[26] VanderLaan DP, Forrester DL, Petterson LJ, Vasey PL. The prevalence of *fa’afafine* relatives among Samoan gynephilic men and *fa’afafine*. Archives of Sexual Behavior. 2013;42(3):353–9. doi: 10.1007/s10508-012-0015-7 23054259 10.1007/s10508-012-0015-7

[27] VanderLaan DP, Vokey JR, Vasey PL. Is transgendered male androphilia familial in non-Western populations? The case of a Samoan village. Archives of Sexual Behavior. 2013;42(3):361–70. doi: 10.1007/s10508-012-0037-1 23187702 10.1007/s10508-012-0037-1

[28] Laumann EO. The social organization of sexuality: Sexual practices in the United States Chicago (IL): University of Chicago Press; 1994.

[29] Bailey JM, Vasey PL, Diamond LM, Breedlove SM, Vilain E, Epprecht M. Sexual orientation, controversy, and science. Psychological Science in the Public Interest. 2016;17(2):45–101. doi: 10.1177/1529100616637616 27113562 10.1177/1529100616637616

[30] Danver SL. Native peoples of the world: An encyclopedia of groups, cultures and contemporary issues Armonk (NY): Routledge; 2015.

[31] Comisión Nacional para el Desarrollo de los Pueblos Indígenas. Regiones indígenas de México. Mexico City: Impresora y Encuadernadora Progreso, S.A. de C.V.; 2006. Available from: http://www.cdi.gob.mx/regiones/regiones_indigenas_cdi.pdf

[32] Instituto Nacional de Estadística y Geografía. Perfil sociodemográfico de la población que habla lengua indígena. 2009. Available from: http://internet.contenidos.inegi.org.mx/contenidos/Productos/prod_serv/contenidos/espanol/bvinegi/productos/censos/poblacion/poblacion_indigena/leng_indi/PHLI.pdf

[33] Consejo Nacional de Población. Proyecciones de la población de México 2010–2050. 2016. Available from: http://www.conapo.gob.mx/es/CONAPO/Proyecciones_Datos

[34] Miano Borruso M. Hombres, mujeres y muxe en la sociedad zapoteca del Istmo de Tehuantepec [dissertation]. Mexico City: Escuela Nacional de Antropología e Historia; 1999.

[35] Chiñas B. Isthmus Zapotec attitudes toward sex and gender anomalies In: Murray SO, editor. Latin America male homosexualities. Albuquerque: University of New Mexico Press; 1995.

[36] Mirandé A. Hombres mujeres: An indigenous third gender. Men and Masculinities. 2016;19: 384–409.

[37] Gómez FR., Semenyna SW, Court L, Vasey PL. Recalled separation anxiety in childhood in Istmo Zapotec men, women, and muxes. Archives of Sexual Behavior. 2017;46(1):109–117. doi: 10.1007/s10508-016-0917-x 28050743 10.1007/s10508-016-0917-x

[38] Bowen RC, Offord DR, Boyle MH. The prevalence of overanxious disorder and separation anxiety disorder: Results from the Ontario Child Health Study. Journal of the American Academy of Child & Adolescent Psychiatry. 1990;29(5):753–8. 2228929 10.1097/00004583-199009000-00013

[39] Shear K, Jin R, Ruscio AM, Walters EE, Kessler RC. Prevalence and correlates of estimated DSM-IV child and adult separation anxiety disorder in the National Comorbidity Survey Replication. American Journal of Psychiatry. 2006;163(6):1074–83. doi: 10.1176/appi.ajp.163.6.1074 16741209 10.1176/appi.ajp.163.6.1074 PMC1924723

[40] Bailey JM. The man who would be queen: The science of gender-bending and transsexualism Washington, DC: Joseph Henry Press; 2003.

[41] Lippa RA. Gender‐related traits in gay men, lesbian women, and heterosexual men and women: The virtual identity of homosexual‐heterosexual diagnosticity and gender diagnosticity. Journal of Personality. 2000;68(5):899–926. 11001153 10.1111/1467-6494.00120

[42] Lippa RA. Gender-related traits of heterosexual and homosexual men and women. Archives of Sexual Behavior. 2002;31(1):83–98. 11910795 10.1023/a:1014035302843

[43] Lippa RA. Sex differences and sexual orientation differences in personality: Findings from the BBC internet survey. Archives of Sexual Behavior. 2008;37(1):173–87. doi: 10.1007/s10508-007-9267-z 18074219 10.1007/s10508-007-9267-z

[44] Zheng L, Lippa RA, Zheng Y. Sex and sexual orientation differences in personality in China. Archives of Sexual Behavior. 2011;40(3):533–41. doi: 10.1007/s10508-010-9700-6 21080048 10.1007/s10508-010-9700-6

[45] Consulado de Carrera de México en Leamington. Visitors who do not require a visa, with a stay of up to 180 days. 2016. Available from: https://consulmex.sre.gob.mx/leamington/index.php/non-mexicans/visas/111-visitor-visa

[46] Kinsey AC, Pomeroy WB, Martin CE. Sexual behavior in the human male Philadelphia (PA): W.B. Saunders; 1948 10.2105/ajph.93.6.894 PMC1447861 12773346

[47] Cohen J. Statistical power analysis for the behavioral sciences (2nd ed.). Hillsdale (NJ): Lawrence Erlbaum Associates Inc.; 1977.

[48] Fritz CO, Morris PE, Richler JJ. Effect size estimates: Current use, calculations, and interpretation. Journal of Experimental Psychology: General. 2012;141(1):2. 21823805 10.1037/a0024338

[49] Pattatucci AML. Molecular investigations into complex behavior: Lessons from sexual orientation studies. Human Biology. 1998;70(2):367–86. 9549244

[50] Zucker KJ, Lawrence AA. Epidemiology of gender identity disorder: Recommendations for the standards of care of The World Professional Association for Transgender Health. International Journal of Transgenderism. 2009;11(1):8–18.

[51] Arcelus J, Bouman WP, Van Den Noortgate W, Claes L, Witcomb G, Fernandez-Aranda F. Systematic review and meta-analysis of prevalence studies in transsexualism. European Psychiatry. 2015;30(6):807–15. doi: 10.1016/j.eurpsy.2015.04.005 26021270 10.1016/j.eurpsy.2015.04.005

[52] Zucker KJ. Epidemiology of gender dysphoria and transgender identity. Sexual Health. 2017. doi: https://doi.org/10.1071/SH17067 10.1071/SH17067 28838353

[53] Wilson EO. Sociobiology: The new synthesis Cambridge (MA): Belknap Press; 1975.

[54] Abild ML, VanderLaan DP, Vasey PL. Does geographic proximity influence the expression of avuncular tendencies in Canadian androphilic males? Journal of Cognition and Culture. 2014;14(1–2):41–63.

[55] Bobrow D, Bailey JM. Is male homosexuality maintained via kin selection? Evolution and Human Behavior. 2001;22(5):361–8.

[56] Camperio Ciani A, Battaglia U, Liotta M. Societal norms rather than sexual orientation influence kin altruism and avuncularity in tribal Urak-Lawoi, Italian, and Spanish adult males. The Journal of Sex Research. 2016;53(2):137–48. doi: 10.1080/00224499.2014.993748 26132515 10.1080/00224499.2014.993748

[57] Forrester DL, VanderLaan DP, Parker JL, Vasey PL. Male sexual orientation and avuncularity in Canada: Implications for the kin selection hypothesis. Journal of Cognition and Culture. 2011;11(3–4):339–52.

[58] Rahman Q, Hull MS. An empirical test of the kin selection hypothesis for male homosexuality. Archives of Sexual Behavior. 2005;34(4):461–7. doi: 10.1007/s10508-005-4345-6 16010468 10.1007/s10508-005-4345-6

[59] VanderLaan DP, Petterson LJ, Vasey PL. Elevated kin-directed altruism emerges in childhood and is linked to feminine gender expression in Samoan *fa’afafine*: A retrospective study. Archives of Sexual Behavior. 2017;46(1):95–108. doi: 10.1007/s10508-016-0884-2 27987088 10.1007/s10508-016-0884-2

[60] VanderLaan DP, Vasey PL. Relationship status and elevated avuncularity in Samoan *fa’afafine*. Personal Relationships. 2012;19(2):326–39.

[61] Vasey PL, VanderLaan DP. Materteral and avuncular tendencies in Samoa. Human Nature. 2009;20(3):269–81.

[62] Vasey PL, VanderLaan DP. An adaptive cognitive dissociation between willingness to help kin and non-kin in Samoan *fa’afafine*. Psychological Science. 2010;21(2):292–7. doi: 10.1177/0956797609359623 20424059 10.1177/0956797609359623

[63] Vasey PL, VanderLaan DP. Avuncular tendencies and the evolution of male androphilia in Samoan *fa’afafine*. Archives of Sexual Behavior. 2010;39(4):821–30. doi: 10.1007/s10508-008-9404-3 18810630 10.1007/s10508-008-9404-3

[64] Vasey PL, VanderLaan DP. Monetary exchanges with nieces and nephews: a comparison of Samoan men, women, and *fa'afafine*. Evolution and Human Behavior. 2010;31(5):373–80.

[65] VanderLaan DP, Ren Z, Vasey PL. Male androphilia in the ancestral environment. Human Nature. 2013;24(4):375–401. doi: 10.1007/s12110-013-9182-z 24091924 10.1007/s12110-013-9182-z

[66] Ciani AC, Pellizzari E. Fecundity of paternal and maternal non-parental female relatives of homosexual and heterosexual men. PloS one. 2012;7(12):e51088 doi: 10.1371/journal.pone.0051088 23227237 10.1371/journal.pone.0051088 PMC3515521

[67] Iemmola F, Ciani AC. New evidence of genetic factors influencing sexual orientation in men: Female fecundity increase in the maternal line. Archives of Sexual Behavior. 2009;38(3):393–9. doi: 10.1007/s10508-008-9381-6 18561014 10.1007/s10508-008-9381-6

[68] King M, Green J, Osborn DP, Arkell J, Hetherton J, Pereira E. Family size in white gay and heterosexual men. Archives of Sexual Behavior. 2005;34(1):117–22. doi: 10.1007/s10508-005-1006-8 15772775 10.1007/s10508-005-1006-8

[69] Semenyna SW, Petterson LJ, VanderLaan DP, Vasey PL. A comparison of the reproductive output among the relatives of Samoan androphilic *fa’afafine* and gynephilic men. Archives of Sexual Behavior. 2017;46(1):87–93. doi: 10.1007/s10508-016-0765-8 27785648 10.1007/s10508-016-0765-8

[70] VanderLaan DP, Gothreau LM, Bartlett NH, Vasey PL. Separation anxiety in feminine boys: Pathological or prosocial? Journal of Gay & Lesbian Mental Health. 2010;15(1):30–45.

