# Supplementary material for: Familial patterning and prevalence of male androphilia among Istmo Zapotec men and muxes
Source: PLoS One. 2018 Feb 21;13(2):e0192683. doi: 10.1371/journal.pone.0192683 (PMC5821324; doi:10.1371/journal.pone.0192683)
Supplement: S1 Appendix — (DOCX) [file pone.0192683.s001.docx]

**Questions about your father’s side of the family (Paternal Relatives)**

1) How many sons did your father’s mother give birth to?

2) How many of these sons were muxes?

3) How many daughters did your father’s mother give birth to?

**Please provide the following information about each of your father’s mother’s biological children (your aunts and uncles on your father’s side of the family who were not adopted), from the first born to the last born:**

1) Whether that person is your father, aunt, or uncle (if you do not know the order, please indicate your oldest uncle, oldest aunt, youngest brother, and youngest aunt if possible).

2) How many sons that person had?

3) How many of those sons were muxe?

4) How many daughters that person had?

5) Please list whether the first born was a boy, girl, or muxe.

6) The country or countries the children were born in (if there are no children, please list where your aunt or uncle live).

| Father/Uncle/Aunt | # Sons | # Muxes | # Daughters | Oldest Boy, Girl, or Muxe? | Place of Birth |
| --- | --- | --- | --- | --- | --- |
|  |  |  |  |  |  |
|  |  |  |  |  |  |
|  |  |  |  |  |  |
|  |  |  |  |  |  |
|  |  |  |  |  |  |
|  |  |  |  |  |  |
|  |  |  |  |  |  |
|  |  |  |  |  |  |
|  |  |  |  |  |  |
|  |  |  |  |  |  |
|  |  |  |  |  |  |

**Questions about your mother’s side of the family (Paternal Relatives)**

1) How many sons did your mother’s mother give birth to?

2) How many of these sons were muxes?

3) How many daughters did your mother’s mother give birth to?

**Please provide the following information about each of your mother’s mother’s biological children (your aunts and uncles on your father’s side of the family who were not adopted), from the first born to the last born:**

1) Whether that person is your mother, aunt, or uncle (if you do not know the order, please indicate your oldest uncle, oldest aunt, youngest brother, and youngest aunt if possible).

2) How many sons that person had?

3) How many of those sons were muxes?

4) How many daughters that person had?

5) Please list whether the first born was a boy, girl, or muxe.

6) The country or countries the children were born in (if there are no children, please list where your aunt or uncle live).

| Mother/Uncle/Aunt | # Sons | # Muxe | # Daughters | Oldest Boy, Girl, or Muxe? | Place of Birth |
| --- | --- | --- | --- | --- | --- |
|  |  |  |  |  |  |
|  |  |  |  |  |  |
|  |  |  |  |  |  |
|  |  |  |  |  |  |
|  |  |  |  |  |  |
|  |  |  |  |  |  |
|  |  |  |  |  |  |
|  |  |  |  |  |  |
|  |  |  |  |  |  |
|  |  |  |  |  |  |
|  |  |  |  |  |  |
