# Supplementary material for: Familial patterning and prevalence of male androphilia among Istmo Zapotec men and muxes
Source: PLoS One. 2018 Feb 21;13(2):e0192683. doi: 10.1371/journal.pone.0192683 (PMC5821324; doi:10.1371/journal.pone.0192683)
Supplement: S2 Appendix — (DOCX) [file pone.0192683.s002.docx]

**Preguntas sobre la familia del lado de su padre (Parientes Paternos)**

1) ¿A cuántos hijos la madre de su padre le dio luz?

2) ¿Cuántos de estos hijos era muxes?

3) ¿A cuántas hijas la madre de su padre le dio luz?

**Por favor provee la siguiente información sobre cada uno de los/as hijos/as biológicos/as de la madre de su padre (los tíos y tías de la familia del lado de su padre que no furon adoptados/as), desde el primero al último nacido/primera a la última nacida.**

1) Indique si esa persona es su padre, tío o tía (si no conoce el orden, por favor indique su tío mayor, tía mayor, tío menor y tía menor si es posible).

2) ¿Cuántos hijos tuvo esa persona?

3) ¿Cuántos de esos hijos eran muxes?

4) ¿Cuántas hijas tuvo esa persona?

5) Por favor si el/la mayor es nene, nena o muxe.

6) El país o países en donde los/as niños/as nacieron (si no hay niños/as, por favor indique donde vive su tío o tía).

| Padre/Tío/Tía | # Hijos | # Muxes | # Hijas | ¿Nene, Nena o Muxe? | Lugar de Nacimiento |
| --- | --- | --- | --- | --- | --- |
|  |  |  |  |  |  |
|  |  |  |  |  |  |
|  |  |  |  |  |  |
|  |  |  |  |  |  |
|  |  |  |  |  |  |
|  |  |  |  |  |  |
|  |  |  |  |  |  |
|  |  |  |  |  |  |
|  |  |  |  |  |  |
|  |  |  |  |  |  |
|  |  |  |  |  |  |

**Preguntas sobre la familia del lado de su madre (Parientes Maternos)**

1) ¿A cuántos hijos la madre de su madre le dio luz?

2) ¿Cuántos de estos hijos era muxes?

3) ¿A cuántas hijas la madre de su madre le dio luz?

**Por favor provee la siguiente información sobre cada uno de los/as hijos/as biológicos/as de la madre de su madre (los tíos y tías de la familia del lado de su madre que no fueron adoptados/as), desde el primero al último nacido/primera a la última nacida.**

1) Indique si esa persona es su madre, tío o tía (si no conoce el orden, por favor indique su tío mayor, tía mayor, tío menor y tía menor si es posible).

2) ¿Cuántos hijos tuvo esa persona?

3) ¿Cuántos de esos hijos eran muxes?

4) ¿Cuántas hijas tuvo esa persona?

5) Por favor si el/la mayor es nene, nena o muxe.

6) El país o países en donde los/as niños/as nacieron (si no hay niños/as, por favor indique donde vive su tío o tía).

| Madre/Tío/Tía | # Hijos | # Muxes | # Hijas | ¿Nene, Nena o Muxe? | Lugar de Nacimiento |
| --- | --- | --- | --- | --- | --- |
|  |  |  |  |  |  |
|  |  |  |  |  |  |
|  |  |  |  |  |  |
|  |  |  |  |  |  |
|  |  |  |  |  |  |
|  |  |  |  |  |  |
|  |  |  |  |  |  |
|  |  |  |  |  |  |
|  |  |  |  |  |  |
|  |  |  |  |  |  |
|  |  |  |  |  |  |
